# Transcatheter tricuspid valve-in-valve replacement in two patients with Ebstein anomaly: technical considerations

**DOI:** 10.1007/s00392-020-01756-0

**Published:** 2020-11-09

**Authors:** Kay Kronberg, Malena Horn, Fritz Mellert, Albrecht Elsässer

**Affiliations:** 1 Klinikum Oldenburg - Universitätsklinik für Innere Medizin - Kardiologie, Oldenburg, Germany; 2 Klinikum Oldenburg - Universitätsklinik für Herzchirurgie, Oldenburg, Germany

## Abstract

**Electronic supplementary material:**

The online version of this article (10.1007/s00392-020-01756-0) contains supplementary material, which is available to authorized users.

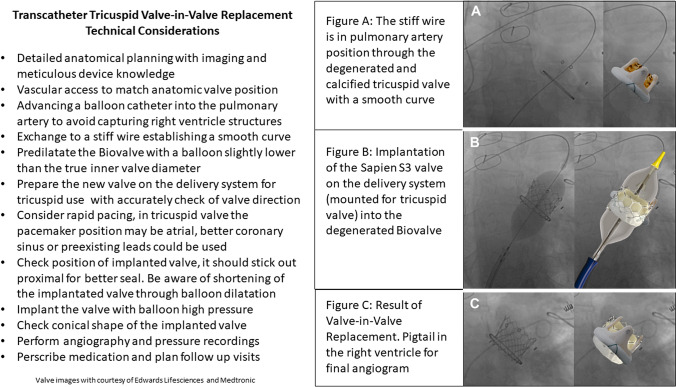

Sirs:

In patients with Ebstein anomaly, the tricuspid valve leaflets are attached to the walls and the septum of the right ventricle. This often leads to tricuspid regurgitation so that in some patients, surgical valve replacement has to be performed. Particularly in young patients, an implanted bioprosthesis bare the risk of early degeneration und dysfunction. It is noteworthy that in many cases, a redo operation bears a high risk of mortality and morbidity. Therefore, transcatheter tricuspid valve-in-valve implantation (TVIV) can be an alternative to a redo surgery, especially in patients with complex cardiac anatomy [1, 3–6, 9, 10].

In contrast to the common valve-in-valve therapy in aortic position, there are only a few results for the tricuspid position, but reported outcomes are often excellent. Most of the cases show impressive improvement concerning the measured mean gradient and the clinical status. Problems like tricuspid valve dysfunction, endocarditis or leafletthrombosis are uncommon after a valve-in-valve therapy [1, 10].

We report our experience of TVIV in two patients with Ebstein anomaly. The first patient (patient 1) is a 57-year-old man who presented in NYHA class III with recurrent dizziness, shortness of breath and oedema. A 2/6 diastolic murmur was audible, the electrocardiogram documented a sinus rhythm. He had a 33 mm HANCOCK II (MEDTRONIC, Minneapolis) tricuspid valve replacement at the age of 39 years. Echocardiography showed a degenerated and heavily sclerosed HANCOCK II prosthesis with an elevated mean pressure gradient of 8 mmHg (Fig. 1b, video 2). Computed tomography (CT) revealed a massive right atrial dilatation (Fig. 1a, b, video 1). With the age of 48 years, the patient developed a third degree AV block and received a pacemaker. Because of his tricuspid bioprosthesis, epicardial electrodes had to be utilized. On admission, the pacemaker test showed battery depletion.

**Fig. 1:**
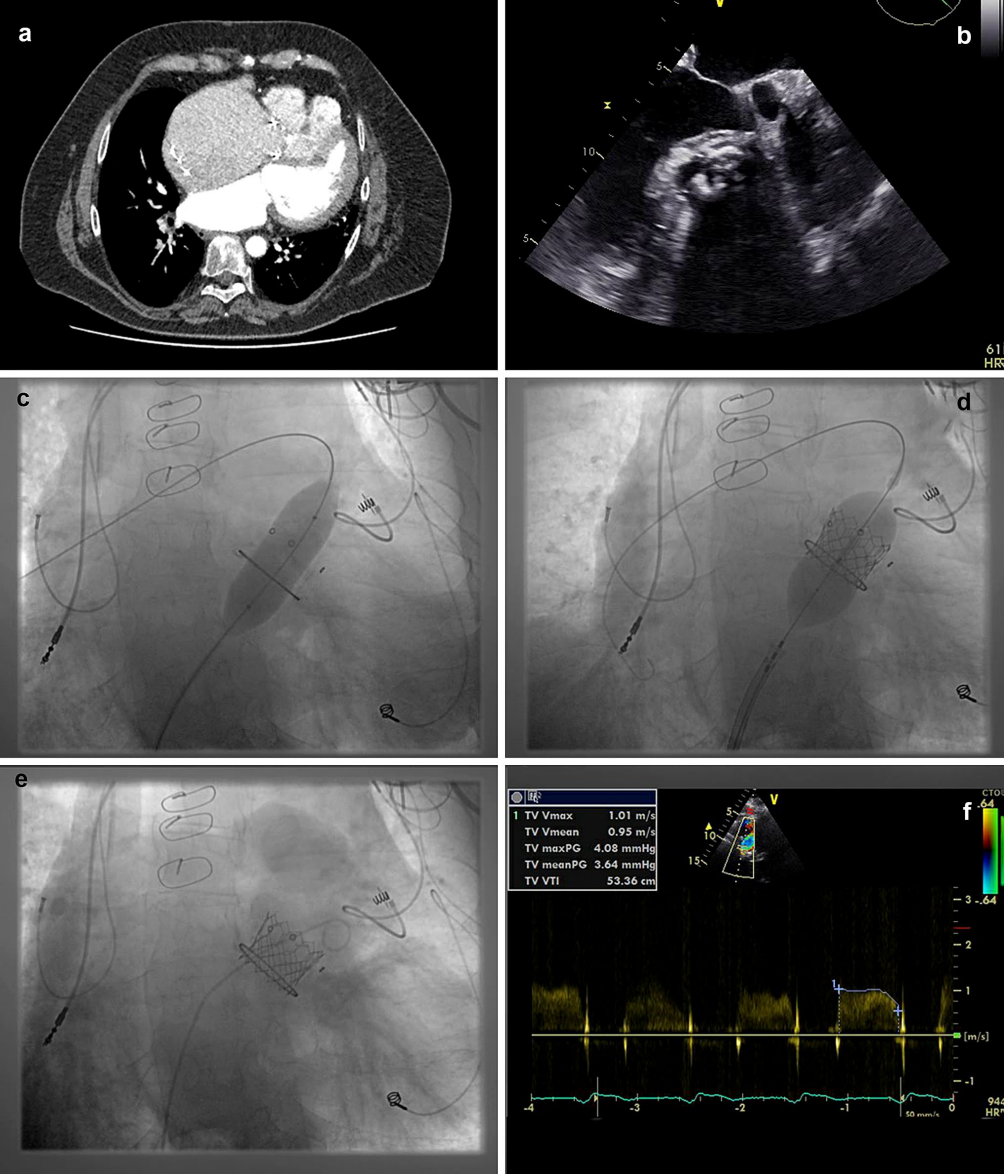
CT, transesophageal echocardiography, valve placement and final Doppler

The second patient (patient 2) is a 34-year-old male with Ebstein anomaly and Wolff–Parkinson–White syndrome. He received an ablation therapy of an accessory pathway at the age of 22 years. Two years later, a Carpentier-Edwards PERIMOUNT bioprosthesis (Edwards Lifesciences Corporation; Irvine, Calif) was implanted due to severe tricuspid insufficiency. He presented with shortness of breath and oedema in NYHA class III. On transthoracic echocardiography, the PERIMOUNT bioprosthesis was heavily degenerated and sclerosed (Fig. 2a) with a breath depending mean gradient of 10 to 14 mmHg across the valve (Fig. 2b).

**Fig. 2:**
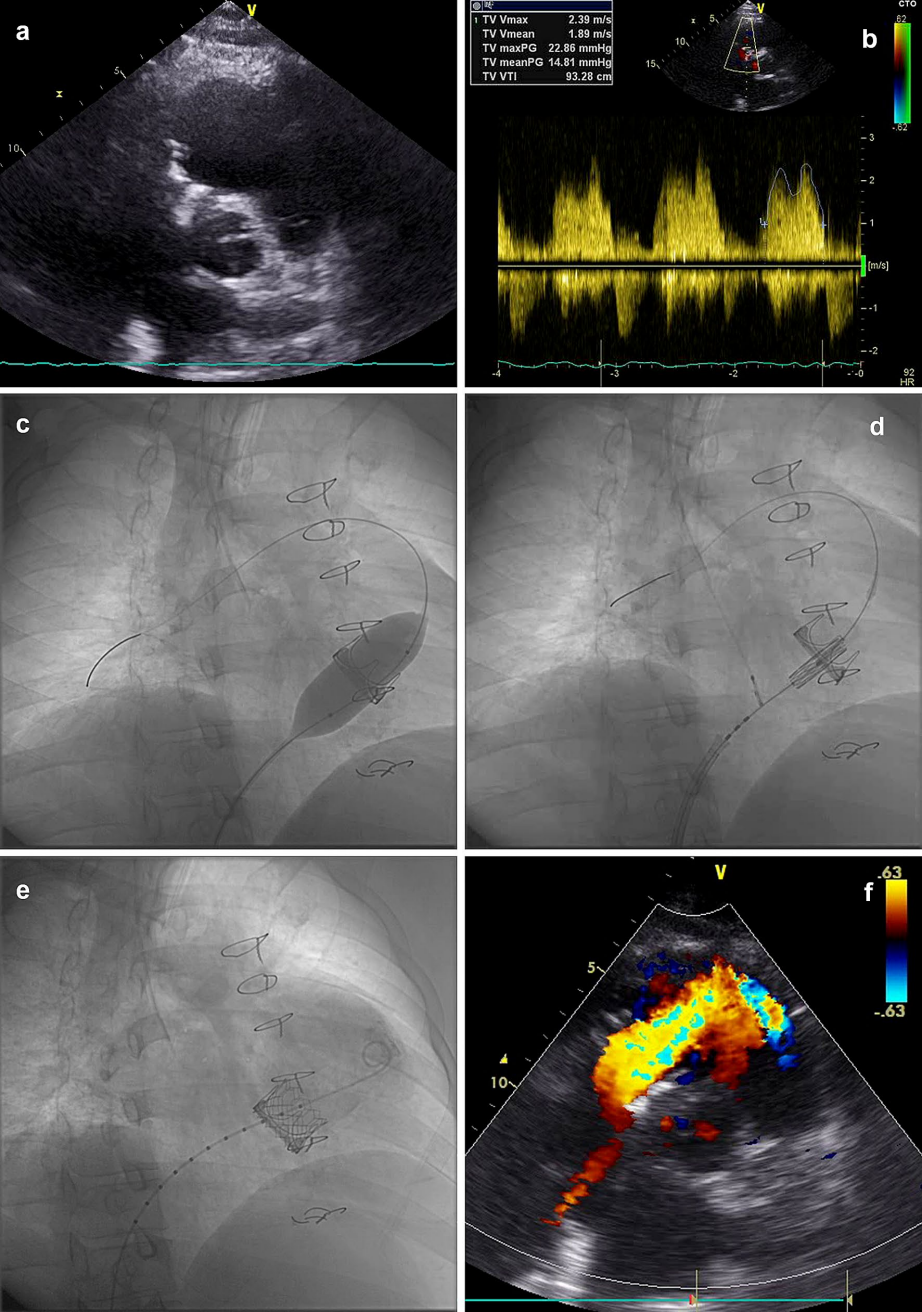
TTE, Doppler, valve placement and final colour Doppler

For the discussion of the treatment options, all available imaging studies were revisited in the heart team (CT, echocardiography in both patients and additional invasive monitoring in patient 1).

The 33 mm HANCOCK II valve (Medtronic) had an outer diameter of 33 mm and a true inner diameter of 30 mm, it was heavily sclerosed (Fig. 1a, b, video 3). The 33 mm PERIMOUNT bioprosthesis (Edwards Lifesciences) in patient 2 had an outer diameter of 33 mm and a true inner diameter of 28.5 mm.

Due to these sizes, the Edwards SAPIEN 3 Transcatheter Heart Valve (Edwards Lifesciences Corporation; Irvine, Calif) with an outer diameter of 29–29.5 mm and an expanded height of 22.5 mm seemed suitable for both patients [2, 7, 8].

Additionally, it seems that Ebstein patients with surgically implanted tricuspid bioprosthesis have a favourable anatomic position for transcatheter valve-in-valve implantation. In our patient 1, the prosthetic valve ring showed a 45° angle to body centre line; in patient 2, we found a 40° angle (Figs. 1a, c, 2c). On possible explanation might be the significantly enlarged right atrium and the need of sewing the initial tricuspid prosthetic valve to the fibrous part of the septum. This anatomical situation and the high risk of reoperation in Ebstein anomaly encouraged our decision towards a tricuspid valve-in-valve procedure [1, 4–9, 10].

After for both patients informed consent has been obtained, we started the intervention in our hybrid operating room in general anaesthesia. Because of the 40–45° valve angles, we choose the right femoral vein for vascular access. After placing a short 6F sheath, we passed a balloon wedge pressure catheter 110/6F (Arrow Medical, Kington, UK) through the sclerosed tricuspid valve into the pulmonary artery. Using a floating catheter, we avoided to capture the existing right ventricular structures. Hemodynamic values were taken, whereas the invasively measured mean gradient was 8 mmHg in patient 1 and 12 mmHg in patient 2. On fluoroscopy, the massive valve calcification in the first patient could be observed (video 3).

To ensure a good wire support for balloon angioplasty, we placed an Amplatzer extra Stiff Wire 260 (Cook Medical Inc.; Bloomington, Ind) in patient 1 and a Lunderquist 300 wire (Cook Medical Inc.) in patient 2 through the wedge pressure catheter. With a 28 mm Cristal Balloon Catheter 28/50/110 (Balt, USA, Medical Devices, Irvine, CA), we predilated the tricuspid bioprosthesis too fully balloon expansion in both patients (Figs. 1c, 2c, video 4). The position of the balloon was stable during the cardiac cycle.

After preparation, the SAPIEN 3 valve system could be gently advanced over the stiff wire (Fig. 2d). Due to the favourable angle of the implantated valves in both Ebstein patients, the orthogonal alignment of the wire with the ring of the bioprosthesis was given (Figs. 1c, d, 2c, d).

To guarantee a stable SAPIEN position throughout the heart beat cycle, we needed in the first patient no rapid pacing due to the heavily calcified prosthesis. In the second patient, we used atrial pacemaker overstimulation at a rate of 140/min to minimize heart movement during implantation (Electrode BP2502-10 Biosensors international, Singapore), (Fig. 2c, d, f, video 5). Alternatively, a deflectable pacemaker electrode could have been positioned over the coronary sinus in middle cardiac vein to realize more rapid ventricular stimulation in patients with tricuspid prosthesis [3].

To ensure an appropriate position, the SAPIEN 3 prosthesis has to be placed about 10% above the sewing ring of the implanted valve. The proximal portion of the 29 mm SAPIEN 3 valve is wrapped with a relatively long inner skirt (11.6 mm) and a smaller outer skirt (8.1 mm) of polyethylene terephthalate (PET) for sealing. The foreshortening while implantation is mainly on the inflow side and around 8.5 mm from a crimped height of 31 mm to expanded height of 22.5 mm. After implantation with this precaution, the cobalt chromium stent of the SAPIEN 3 lies minimally proximal to the preexisting valve on fluoroscopy. This way the outer skirt of the SAPIEN 3 valve seals up at best to the sewing ring of the existing valve [2, 7, 8]. For a save and complete sealing, the SAPIEN 3 valve received a final balloon dilatation and a conical shape of the SAPIEN 3 stent could be seen (Figs. 1e, 2e, f, video 6).

We ended the intervention with a competent valve and a mean gradient of 4 mmHg in patient 1 and of 5 mmHg for patient 2. The total fluoroscopy time was 12.3 min for patient 1 and 15.9 min for patient 2. The procedure time was 80 min and 75 min. The pacemaker device in patient 1 has been replaced in the same narcosis. Both patients recovered well and showed good valve function with no apparent valve leakage on echocardiography (Figs. 1f, 2f, video 7).

Leaflet thrombosis is an uncommon but important complication after valve-in-valve therapy, especially in tricuspid position, in which risks of bioprosthetic valve thrombosis is higher than in other positions not depending on the implanted valve type [10, 4]. This concern is even increased due to Ebstein anomaly, most likely caused by the right atrium and RV enlargement and abnormal RV diastolic hemodynamics [10].

Given these conditions, it is worth considering the use of anticoagulant or dual antiplatelet therapy rather than a single antiplatelet agent. There are approximately half of Ebstein patients with TVIV treated with ASS as antiplatelet therapy and the other half additionally with anticoagulants like coumarin derivates [1, 4, 10].

Our 57-year-old patient 1 could be discharged 5 days after the procedure. Due to intermitting atrial fibrillation, the discharged therapy was ASS 100 mg and Edoxaban 60 mg. Our 34-year-old patient 2 was discharged on the fourth day after the procedure. He received a combination of ASS 100 mg and Clopidogrel 75 mg for 3 months. Endocarditis prophylaxis and ASS 100 mg should be prescribed lifelong [4].

On follow-up, there was no stenosis and no valve insufficiency on echocardiography in our two patients. They both had good RV functions and were in a stable condition with no oedema. After 9 months, the mean gradient was 3 mmHg in patient 1 (video 8) and after 3 months, the mean gradient was 5 mmHg in patient 2 (video 9).

Mid-term valve-related outcomes after transcatheter tricuspid valve-in-valve replacement are promising in a data collection of TVIV 306 cases [1] and in 81 Ebstein patients [10].

According to our experience, the procedure is technically feasible with good results but requires detailed anatomical planning and meticulous device knowledge [2, 7].

## Electronic supplementary material

Below is the link to the electronic supplementary material.

Supplementary Video 1 (WMV 465 kb)

Supplementary Video 2 (AVI 938 kb)

Supplementary Video 3 (WMV 168 kb)

Supplementary Video 4 (AVI 487 kb)

Supplementary Video 5 (WMV 1395 kb)

Supplementary Video 6 (ASF 1364 kb)

Supplementary Video 7 (WMV 465 kb)

Supplementary Video 8 (WMV 458 kb)

Supplementary Video 9 (AVI 723 kb)
